# Poly-ICLC, a TLR3 Agonist, Induces Transient Innate Immune Responses in Patients With Treated HIV-Infection: A Randomized Double-Blinded Placebo Controlled Trial

**DOI:** 10.3389/fimmu.2019.00725

**Published:** 2019-04-09

**Authors:** Mansi Saxena, Rachel L. Sabado, Melissa La Mar, Hiroshi Mohri, Andres M. Salazar, Hanqing Dong, Joel Correa Da Rosa, Martin Markowitz, Nina Bhardwaj, Elizabeth Miller

**Affiliations:** ^1^Icahn School of Medicine at Mount Sinai, New York, NY, United States; ^2^Aaron Diamond AIDS Research Center, Rockefeller University, New York, NY, United States; ^3^Oncovir, Inc., Washington, DC, United States; ^4^Laboratory of Investigative Dermatology, The Rockefeller University, New York, NY, United States

**Keywords:** HIV-1, vaccine, adjuvant, poly-ICLC, toll-like receptor ligand

## Abstract

**Objective:** Toll-like receptor-3 agonist Poly-ICLC has been known to activate immune cells and induce HIV replication in pre-clinical experiments. In this study we investigated if Poly-ICLC could be used for disrupting HIV latency while simultaneously enhancing innate immune responses.

**Design:** This was a randomized, placebo-controlled, double-blinded trial in aviremic, cART-treated HIV-infected subjects. Participants (*n* = 15) were randomized 3:1 to receive two consecutive daily doses of Poly-ICLC (1.4 mg subcutaneously) vs. placebo. Subjects were observed for adverse events, immune activation, and viral replication.

**Methods:** Besides primary outcomes of safety and tolerability, several longitudinal immune parameters were evaluated including immune cell phenotype and function via flowcytometry, ELISA, and transcriptional profiling. PCR assays for plasma HIV-1 RNA, CD4^+^ T cell-associated HIV-1 RNA, and proviral DNA were performed to measure HIV reservoirs and latency.

**Results:** Poly-ICLC was overall safe and well-tolerated. Poly-ICLC-related adverse events were Grade 1/2, with the exception of one Grade 3 neutropenia which was short-lived. Mild Injection site reactions were observed in nearly all participants in the Poly-ICLC arm. Transcriptional analyses revealed upregulation of innate immune pathways in PBMCs following Poly-ICLC treatment, including strong interferon signaling accompanied by transient increases in circulating IP-10 (CXCL10) levels. These responses generally peaked by 24–48 h after the first injection and returned to baseline by day 8. CD4^+^ T cell number and phenotype were unchanged, plasma viral control was maintained and no significant effect on HIV reservoirs was observed.

**Conclusions:** These finding suggest that Poly-ICLC could be safely used for inducing transient innate immune responses in treated HIV^+^ subjects indicating promise as an adjuvant for HIV therapeutic vaccines.

**Trial Registration:** www.ClinicalTrials.gov, identifier: NCT02071095.

## Introduction

Innate immune dysregulation during HIV infection hinders the formation of anti-HIV adaptive immunity (1–6) resulting in rampant viral dissemination and progression to AIDS. Adherence to combination anti-retroviral therapy (cART) regimens controls viremia, restores CD4^+^T cell counts and reverses immune dysfunction to a larg extent. However, cART fails to eradicate latent viral reservoirs, posing a major barrier to achieving sterilizing cure (7). Thus, safe and effective adjuvants that stimulate innate immune responses (8) and reactivate latent HIV, allowing for killing of infected cells, are likely to be a vital element of successful therapeutic immunization strategies in aviremic patients (9).

Pattern recognition receptors, such as Toll-like receptors (TLRs), are key activators of innate immunity. These germ line encoded receptors induce rapid inflammation in response to a wide range pathogen associated molecular patterns (PAMPs) (10). Of the 10 TLRs expressed in humans, TLRs 1,2,4,5,6, and 10 are expressed on the cell surface and recognize pathogenic cell membrane components such as lipoproteins, peptidoglycans, flagellin, etc. TLR 3, TLR 7/8, and TLR9 are intracellular receptors that respond to double stranded RNA, single stranded RNA and unmethylated DNA, respectively. TLR3 is expressed multiple cell types including myeloid dendritic cells (DCs), macrophages, Natural killer cells (NK cells), neuronal cells, fibroblasts, endothelial cells, mast cells, and epithelial cells (11). While TLR7 is also expressed on myeloid DCs, NK cells, macrophages, and mast cells, its expression is enriched in plasmacytoid DCs (pDCs), B cells and T cells (12). TLR9 expression pattern is similar to that of TLR7 as it is also primarily expressed in human memory B cells, NK cells, and pDCs (13). Once activated by their cognate ligands, the TLRs signal through the downstream receptors MyD88 (TLR1, 2, 4, 5, 6, 7, 8, 9) or TRIF (TLRs4 and 3) to initiate a complex signaling cascade that culminates in transcriptional induction of inflammatory genes (14).

TLR agonists are potent immune adjuvants. In general their primary adjuvant effects are orchestrated through activation of immune cells like Natural Killer (NK) cells and antigen presenting cells such as DCs (15). DC maturation, induced by TLR agonists, is marked by up regulation of MHC-I and MHC-II and induction of co-stimulatory markers such CD40, CD80, and CD86. TLR activation also leads to secretion of cytokines in immune cells, such as interleukin (IL) 12 secretion by DCs and interferon-gamma (IFNγ) secretion by NK cells. The cumulative net effect is the induction of T cell activation and other specific or general adaptive immune responses (16). Interestingly, TLR agonists have also been shown to reactivate latent HIV both *ex vivo* (17–19) and in patients (20, 21).

Polyinosinic-polycytidylic acid, and poly-L-lysine (Poly-ICLC) is a double stranded RNA complex that serves as a viral mimic recognized by endosomal receptor TLR3 and cytoplasmic sensors MDA-5 and DHX/DDX RNA helicases (22–24). Its adjuvant effects are multi-faceted, including activation of classical DCs to express high levels of IL-12 and type I IFN (16) to promote Th1 polarization (25). Studies in humanized mice models have validated the significance of Poly-IC as a potent adjuvant for driving DC-induced inflammation and activation of antigen specific cytotoxic T cells (26). Furthermore, Poly-IC has been reported to reverse viral latency in human microglial cells *in vitro* (27).

In clinical trials with healthy volunteers and cancer patients, Poly-ICLC has been found to be overall safe and immunogenic (28–33). Interestingly, Poly-IC has been reported to be more efficient than other TLR ligands at improving immunogenicity and inducing viral control when it is either administered alone (34) or in combination with other components (35–38).

A major challenge in using TLR ligands as therapy during HIV infection is the profound host immune dysfunction induced by the virus, including dampening of TLR responsiveness (6, 39, 40). While viremia suppression by cART has been reported to rescue DC activation (39); whether Poly-ICLC can be safely used as an adjuvant and a latency reversing agent in this setting remains to be determined.

Here we report the results of a randomized, placebo-controlled, double-blinded trial investigating the use of Poly-ICLC in HIV setting (NCT02071095). The primary end point of the study was to establish if Poly-ICLC is safe and well-tolerated in HIV-1-infected subjects on cART. The secondary end points were; (a) to determine whether Poly-ICLC disrupts viral latency in HIV-1-infected individuals on cART and (b) to confirm that Poly-ICLC enhances innate immune responses in HIV-infected subjects on cART, and that its immunostimulatory properties are transient in nature. The secondary endpoints include measuring innate immune activation (DC, NK Cells, soluble factors, and transcriptional responses), and measures of viral RNA and DNA. A special consideration for the use of an immunostimulant during HIV infection is the risk of inducing inappropriate immune activation resulting in increased number of cellular targets of infection. Therefore, multiple parameters of generalized immune activation and exhaustion were monitored as additional safety measures. While we did not observe any clear effects of Poly-ICLC in reversing HIV latency or on the size of viral reservoirs; we did determine that Poly-ICLC was safe and well-tolerated in the HIV-infected population studied. Furthermore, Poly-ICLC lead to transient innate immune stimulation without undue generalized immune activation. These findings suggest that Poly-ICLC may enhance the formation of HIV-specific immune responses when administered with HIV therapeutic vaccines.

## Methods

### Study Design and Eligibility

**Figure 1:**
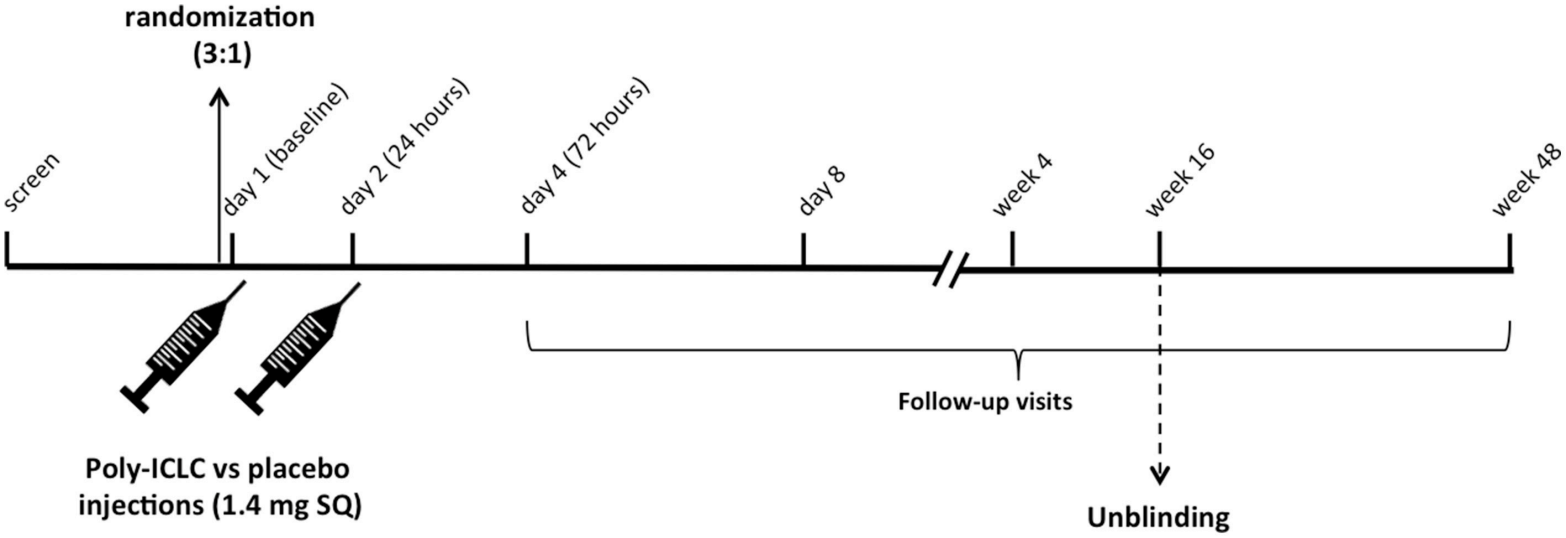
Study Schema. Following screening, eligible subjects are randomized 3:1 to receive Poly-ICLC (1.4 mg SQ) vs. Placebo on days 1 and 2. Participants returned for follow up visits on days 4 and 8, weeks 4, 16, and 48. Unblinding of the study occurred after all subjects completed the week 16 follow up visit as specified by the protocol.

**Figure 2:**
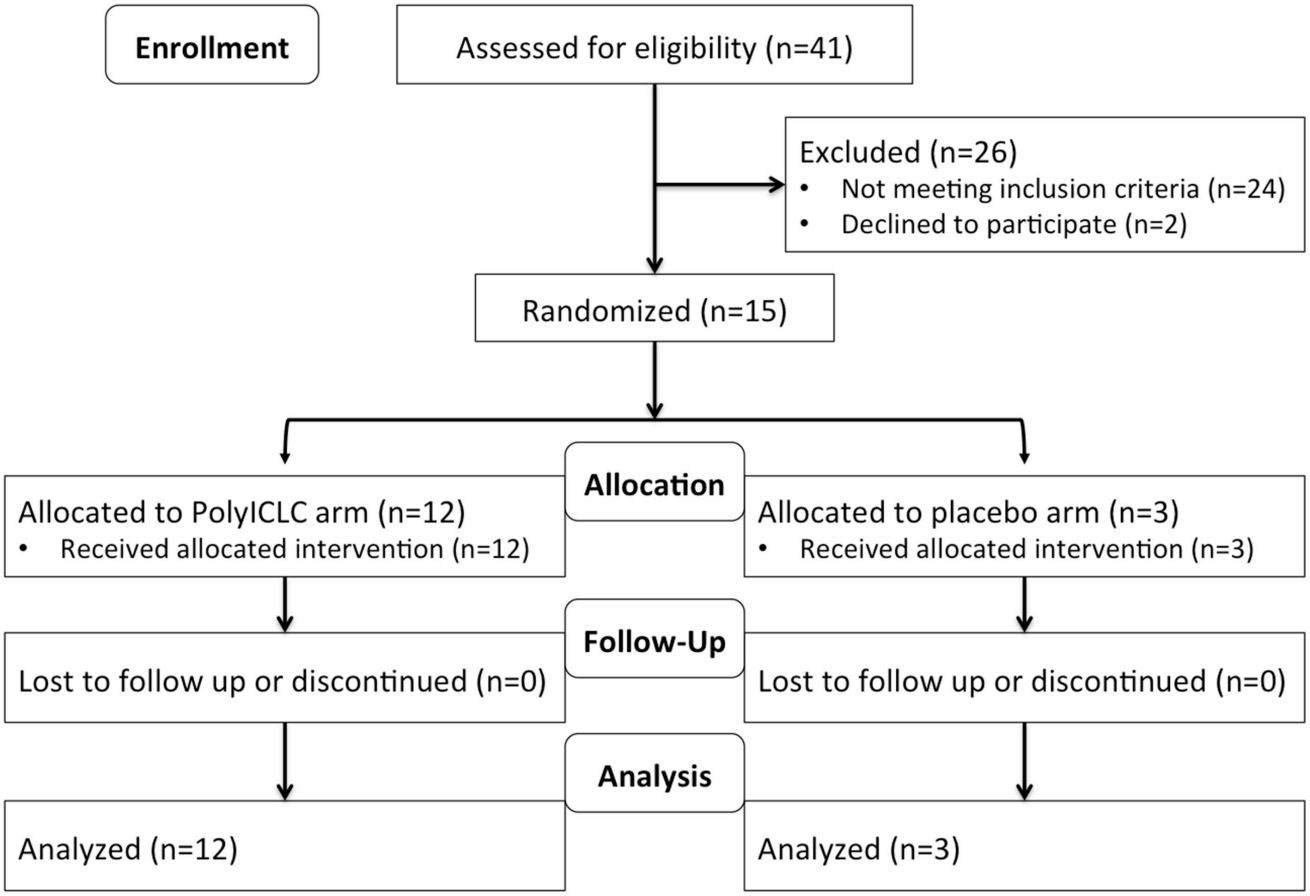
Study flow diagram.

This was a randomized, placebo-controlled, double-blinded, trial in cART-suppressed subjects with HIV infection (NCT02071095). Participants (*N* = 15) were randomized 3:1 to receive 2 consecutive daily doses of Poly-ICLC (Hiltonol®, Oncovir) [1.4 mg subcutaneously (SQ)] vs. placebo (normal saline) (Figure 1) and followed for 48 weeks. Subjects were randomized into blocks of 4 4 8 4. In total 12 participants received Poly-ICLC and 3 participants received the placebo (Figure 2).

Eligible participants included non-pregnant, non-lactating men, and women aged 18–55 years with HIV-infection on cART. Further eligibility was determined based on documented plasma HIV-1 RNA suppression below 50 copies/ml for at least 48 weeks and CD4^+^ T cells >500 cells/mm^3^. Persons with co-morbidities including vascular disease, poorly controlled hypertension, diabetes, hyperlipidemia, renal disease, active hepatitis B/C co-infection, autoimmunity, or multi-drug resistant HIV were excluded. Subjects were recruited from the New York City area from Mount Sinai Hospital Clinics, Rockefeller University Recruitment Office and IRB-approved advertising.

### Plasma HIV-1 RNA Levels

HIV-1 RNA levels in plasma were determined using the Roche COBAS AmpliPrep/COBAS TaqMan HIV-1 Assay, Version 2.0, which detects 10 to 20 × 10^6^ copies per ml.

### DC Enumeration and Activation

To enumerate and assess maturation of CD11c^+^ DCs and pDCs, fresh peripheral blood mononuclear cells (PBMCs) were stained with Live/Dead Violet, HLA-DR PerCP-Cy5.5, CD123 PE-Cy7, Lin1 FITC, CD11c PE-CF594, CD40 APC, CD86 Alexa700, and CD83 PE. Fresh PBMCs, plated for 1 h, were treated with Golgi Plug (1:1,000) for 5 h and stained for surface expression of HLA-DR PerCP-Cy5.5, CD123 PE-Cy7, Lin1 FITC, CD11c PE-CF594, and Live/Dead Violet followed by intracellular cytokine staining with IFNα APC, IL-12 p40/70 PE, and TNFα Alexa700. For the control fluorescence minus one (FMO) wells, the corresponding antibody was omitted from the cocktail. Stained and fixed cells were acquired using BD Fortessa Cytometer.

### NK Cell Enumeration and Activation

Fresh PBMCs were stained with Live/Dead Violet, CD3 PE-CF594, CD16 PerCP-Cy5.5, CD56 BV605, CD69 Alexa700, NKp44 APC, NKG2D PE-Cy7, TIM3 Alexa488, and KIR3DS1/DL1 PE. Stained and fixed cells were acquired using BD Fortessa Cytometer.

### T Cell Phenotype

Thawed PBMCs were stained with CD4 FITC, CD8 Alexa700, HLA-DR PerCP-Cy5.5, CD38 APC, PD-1 BV605, CTLA-4 PE, CD3 PE-CF594, and Live/Dead Violet and acquired using BD Fortessa Cytometer.

### ELISA

Presence of plasma IFNα (PBL Assay Science), IP-10 (R&D systems), sCD14 (R&D systems), TNFα (R&D systems), D-Dimer (Abcam), and C-Reactive Protein (CRP) (Abcam) was evaluated by ELISA per manufacturers protocol.

### Transcriptional Responses

Transcriptional responses profiling 249 inflammation-related genes were evaluated in PBMCs at baseline and days 2, 4, and 8 in a subset of participants (*N* = 12) via NanoString Technologies including statistical analysis (nCounter® gene expression panel, human inflammation kit).

### Quantification of Cell-Associated HIV-1 RNA and DNA in Peripheral CD4^+^T Cells

Cell-associated HIV-1 proviral DNA was measured at baseline and week 4, and cell-associated HIV-1 RNA was measured at each visit through week 16 (baseline, days 2, 4, 8, week 4 and 16). CD4^+^T cells were isolated from PBMCs using Dynabeads FlowComp Human CD4 Kit (Invitrogen). DNA and RNA isolation was performed with AllPrep DNA/RNA Mini Kit (Qiagen). Isolated RNA was treated with DNase I, Amplification Grade (Invitrogen) and purified with RNeasy Mini Kit (Qiagen). Real time PCR was performed with AmpliTaq Gold with Buffer A or AmpliTaq Gold DNA Polymerase with Buffer II and MgCl2 with ROX Reference Dye (Applied Biosystems) using the Stratagene Mx3000P QPCR System (Agilent Technologies). PCR reactions were performed in quadruplicate. Note that for each case an optimal primers/probe set was determined, which gave the highest amplification efficiency among three primer/probe sets tested. Accordingly two primer/probe sets were used, RF/RR/PB (41) for 9 participants and 6F/84R/HIV gag probe (42) for 6 participants.

Cell-associated RNA was primed with random hexamers and reverse transcribed with Superscript II (Invitrogen). PCR reagent mix containing primers, probe, and AmpliTaq Gold was added to each of RT product, and quantitative rtPCR was performed as described above in quadruplicate. No reverse transcriptase control was also included.

### Statistical Methods

Comparisons among the treatment Arms on the proportions of related adverse events was made using Fisher's exact test. Immunologic and virologic parameters between and within treatment Arms were compared at each time points from baseline to post-administration of Poly-ICLC vs. placebo using a linear mixed effect model. Parameters between treatment arms were compared using analysis of covariance, with baseline values as a covariate, after a Box-Cox family variance stabilizing transformation.

For primary end-point of safety, power of Fisher's Exact Test for difference between two proportions when sample sizes are *n1* = 12 and *n2* = 3 was determined (data not shown) and deemed appropriate. For the secondary endpoint, assuming a modest correlation between baseline and follow-up, maximum cellular associated HIV-1 RNA (*r* = 0.32), a sample size of 12 patients in the active arm and 3 patients in the control arm can detect a difference of |2.23| standard deviations in cellular associated HIV-1 RNA maximum fold change between Poly-ICLC SQ and placebo with 2-sided α = 0.05 and power = 92% (calculations from PASS version 12) (43). The assumptions used in this power calculation are consistent with the effect sizes, variability, and correlation over time for the primary outcome in work by Archin et al. (44). Considering the endpoints for cellular phenotype: the sample size provides 80% power at 5% of significance for detecting a minimum difference of |1.95| standard deviations between the two study arms when using a two-sided unpaired *t*-test.

## Results

### Participants and Demographic Details

Participants included 14 males and 1 transgender female with HIV infection. Participants hailed from diverse racial and ethnic backgrounds (40% white, 26.6% black or AA, 6.7% Native American, 6.7% Asian, and 20% multiple or unspecified race, with 46.6% Hispanic or Latino ethnicity). The median age was 39.73 years old (range 26–54), and median baseline CD4^+^ T cell count was 619 cells/mm^3^. A detailed description of baseline patient demographics across the two arms of the study can be found in Table 1.

**Table 1:** Tabulated view of baseline patient demographics in each arm.

		**Arm A: Poly:ICLC**	**Arm B: Placebo**	**Total**
Number of participants		12	3	15
Age (years)	Continuous Mean (Standard Deviation)	41.1 (9.32)	34.33 (4.03)	39.73 (8.61)
Sex (Number)	Female	0	0	0
	Male	12	3	15
Ethnic Category (Number)	Hispanic or Latino	6	1	7
	Not Hispanic or Latino	6	2	8
Racial Categories (Number)	American Indian/Alaska Native	1	0	1
	Asian	1	0	1
	Native Hawaiian or Other Pacific Islander	0	0	0
	Black or African American	4 (1 Hispanic)	0	4
	White	4 (3 Hispanic)	2	6
	More than one race	1 (Hispanic)	0	1
	Unknown/ Unreported	1 (Hispanic)	1 (Hispanic)	2

### Safety and Tolerability

All study participants received both doses of Poly-ICLC or placebo as planned, and all subjects completed visits through week 48 (Figure 2). Overall, Poly-ICLC was safe and well-tolerated with only Grade 1/2 adverse events (AEs) attributed to the study agent, with the exception of one Grade 3 transient neutropenia without clinical sequelae. Injection site reactions (ISR) were the most frequent AE, all of which were Grade 1. Pain was the most common ISR, occurring at 66% of injection sites. Erythema was present at 33% of injection sites. Fever, generally low-grade, was also common (26.6%), lasting 24–48 h. Other frequently reported AEs include chills, myalgias, fatigue, malaise, and headache (Table 2). AEs deemed treatment-related were more common in the Poly-ICLC arm compared with the placebo arm (*P* = 0.012), as was injection site pain (*P* = 0.022). All subjects completed the protocol's dosing regimen, and there were no cases of discontinuation. Two serious adverse events (SAEs) occurred during the study which were deemed unrelated to Poly-ICLC, and both of these occurred in the placebo group.

**Table 2:** Tabulated view of treatment related Adverse Events (AEs).

	**Placebo (*****n=*** **3)**			**Poly:ICLC (*****n=*** **12)**		
	**Grade 1-2**	**Grade 3**	**Grade 4**	**Grade 1-2**	**Grade 3**	**Grade 4**
**GENERAL DISORDERS AND ADMINISTRATION SITE CONDITIONS**						
Chills	0	0	0	3	0	0
Injection site reaction–erythema	0	0	0	5	0	0
Injection site reaction–pain	0	0	0	10	0	0
Fatigue	3	0	0	7	0	0
Fever	0	0	0	4	0	0
Malaise	1	0	0	2	0	0
**NERVOUS SYSTEM DISORDERS**						
Headache	2	0	0	1	0	0
**MUSCULOSKELETAL AND CONNECTIVE TISSUE DISORDERS**						
Myalgias	0	0	0	2	0	0
**HEMATOLOGICAL**						
Low neutrophils count	0	0	0	0	1	0

**Figure 3:**
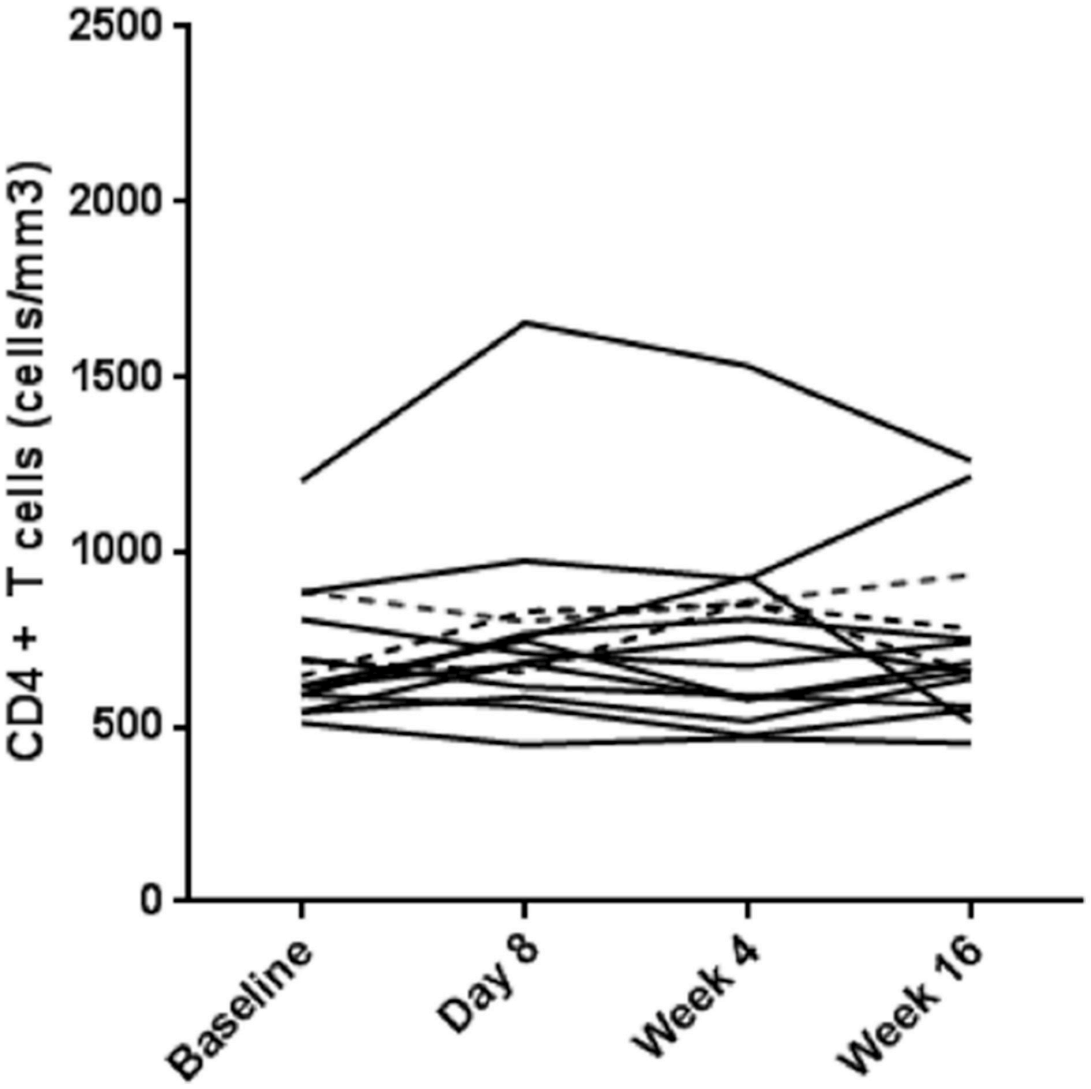
No overall change in CD4^+^ T cells counts in the treatment arm. CD4^+^ T cell counts (cells/mm^3^) for each subject are shown over the course of the study. Values for subjects in Arm A (Poly-ICLC) and Arm B (Placebo) are depicted by solid and dashed lines, respectively.

There was no impact on subjects' CD4^+^ T cell counts following Poly-ICLC injections. The median CD4^+^ T cell count in the Poly-ICLC group increased from 604.5 cells/mm^3^ at baseline to 698 cells/mm^3^ and 656 cells/mm^3^ at day 8 and week 16, respectively, but was not a statistically significant change (Figure 3).

All subjects maintained virologic control with plasma HIV-1 RNA remaining below the limit of detection (<20 copies/ml) throughout the study, with the exception of two discrete measurements of detectable HIV RNA, both in Poly-ICLC arm, with <200 copies/ml. One participant experienced a transient elevation in plasma HIV RNA to 110 copies/ml in the setting of a genital herpes simplex virus outbreak that occurred at their week 4 visit, and another participant was found to have a level of 190 copies/ml that occurred at their week 48 visit (data not shown).

### Immune Responses From PBMCs

**Figure 4:**
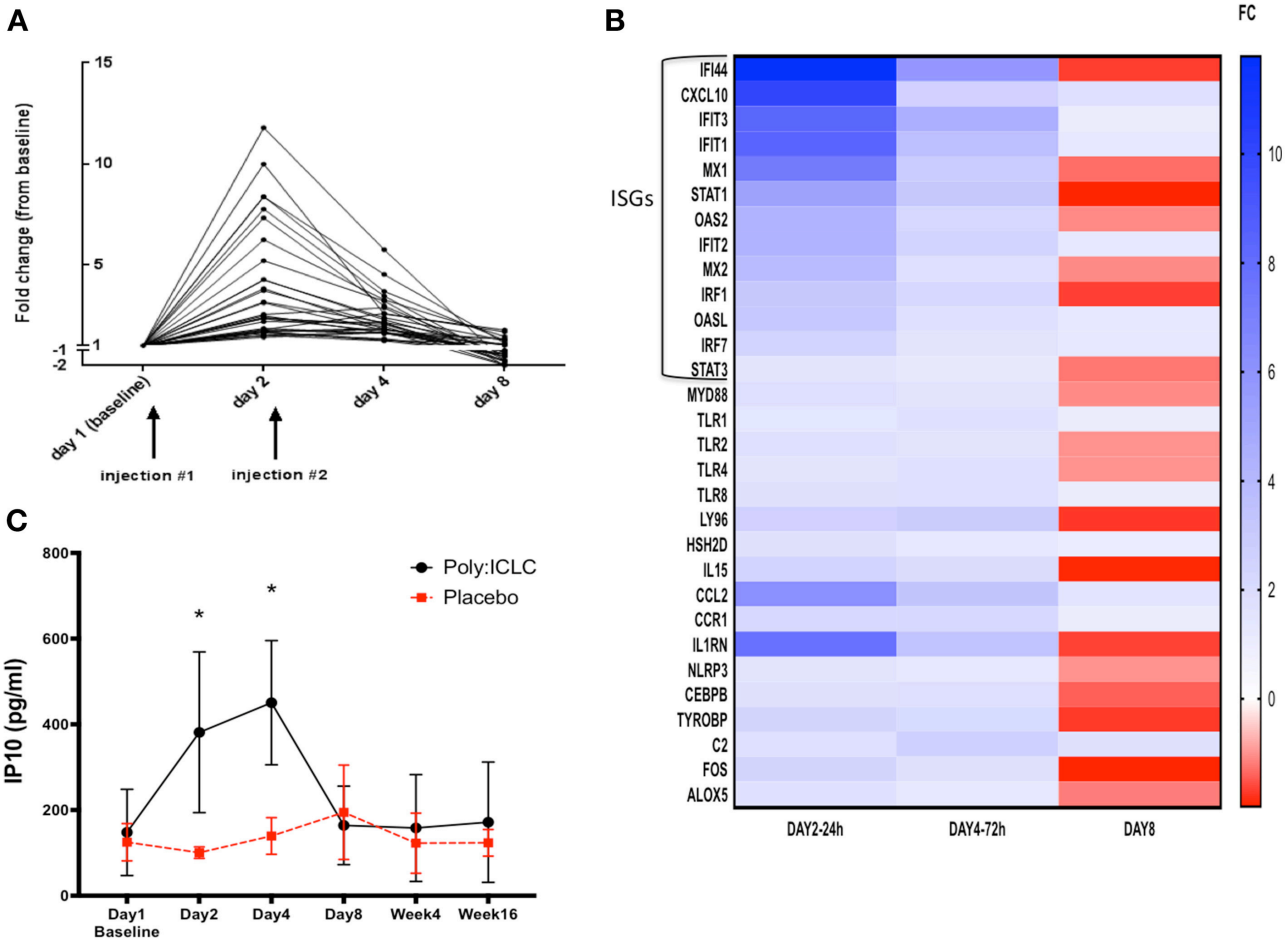
Transient upregulation of pro-inflammatory genes in subjects who received Poly-ICLC, with strong induction of interferon pathway. Transcriptional responses were evaluated longitudinally in subjects' PBMCs using NanoString Technologies (nCounter® gene expression panel, human inflammation kit). **(A)** Fold change (FC) from baseline of significantly upregulated genes from subjects in Arm A (Poly-ICLC) are depicted graphically over time through day 8. Transient upregulation of several genes occurred (*N* = 31), generally peaking at 24 h and returning to baseline shortly thereafter. No significant changes were observed in subjects' PBMCs in Arm B (Placebo) (not shown). **(B)** Heat map of all significantly induced genes in Arm A (Poly-ICLC) represented as a FC over baseline. Most of the highly upregulated genes were found to be interferon-stimulated genes (ISGs) FDR < 1, FC ≥ ±1.5. **(C)** In concordance with strong upregulation of ISGs, plasma levels of IP-10 were transiently upregulated in subjects in Arm A Poly-ICLC (*N* = 12) vs. those in Arm B Placebo (*N* = 3). ^*^p < 0.001.

Transcriptional analyses of PBMCs revealed that multiple innate immune signatures were up regulated in subjects in the Poly-ICLC arm. These responses were transient and generally peaked at 24 h after injection (day 2) and returned to baseline by day 8 (Figure 4A). The interferon pathway was primarily induced, with strong up regulation of interferon-stimulated genes (ISGs) including Interferon Induced proteins with tetratricopeptide repeats (IFITs), interferon regulatory factors (IRFs), and 2′-5′ oligoadenylate synthase (OAS). Up regulation of genes associated with TLR pathways (MyD88, TLR1, TLR2, TLR4, TLR8, and Ly96) (45), T cell and NK cell activation [IL15 and Hematopoietic SH2 Domain Containing (HSH2D)] (46–48) inflammasome activation [NLR Family Pyrin Domain Containing 3 (NLRP3)] (49) and other inflammatory transcription factors, chemokines, and cytokines (FOS, CCL2, CCR1, etc.) was observed to a lesser extent (Figure 4B and Supplemental Table 1). In accordance with the strong up regulation of ISGs found on transcriptional analyses, transient increases in plasma IP-10 in the Poly-ICLC arm were observed on days 2 and 4 compared with baseline and subsequent time points (*p* < 0.001), while no changes were observed in the placebo arm (Figure 4C). Changes in levels of circulating IFNα and other inflammatory markers (IFNβ, TNFα, sCD14, D-dimer, and C reactive protein) were not detected following Poly-ICLC administration, though certain individuals did experience transient increases in D-dimer and CRP that quickly returned to baseline (Supplemental Figure 1).

### T Cell Exhaustion

**Figure 5:**
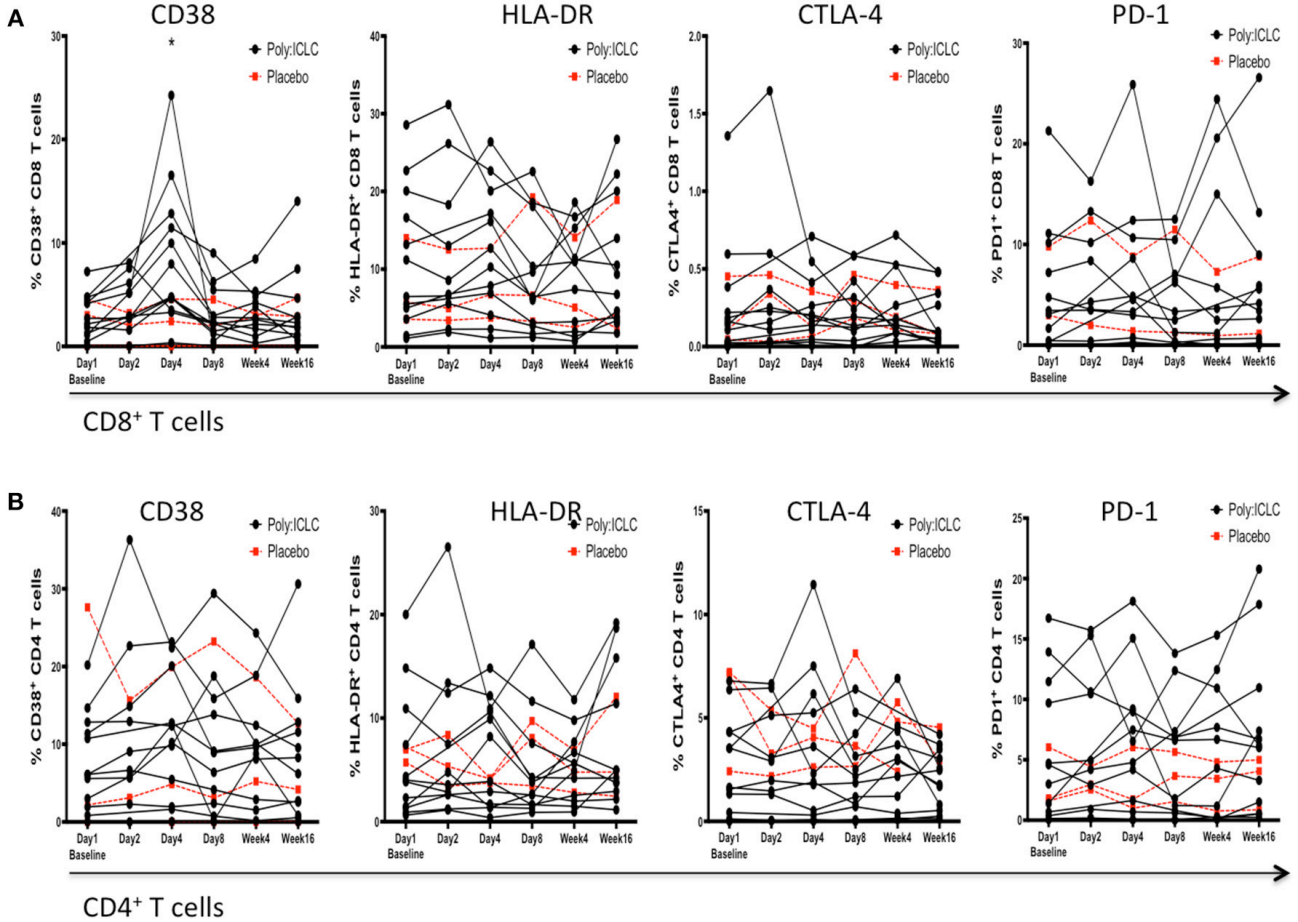
Markers of immune activation and exhaustion on CD4^+^ and CD8^+^ T cells following Poly-ICLC administration. In order to monitor for the secondary induction of generalized immune activation following Poly-ICLC administration, surface expression of CD38, HLA-DR, PD-1, and CTLA-4 was evaluated at each time point via flowcytometry on subjects'; **(A)** CD8^+^ T cells and **(B)** CD4^+^ T cells. No longitudinal changes were found to be statistically significant following Poly-ICLC administration with the exception of transient upregulation of CD38 on CD8^+^ T cells at Day 4. ^*^p < 0.001.

Immune activation on T cells was assessed by surface expression of HLA-DR and CD38. Programmed Cell Death 1 (PD-1) and Cytotoxic T-Lymphocyte Associated Protein 4 (CTLA-4) expression was used as an indicator of T cell exhaustion. Please note that PD1 and CTLA-4 maybe up regulated very early in activated T cells but in later stages are considered markers of exhaustion. Neither HLA-DR nor PD1 and CTLA-4 on CD4^+^ and CD8^+^ T cells was found to be overall changed from baseline following Poly-ICLC administration. However, CD38 was significantly upregulated on day 4 compared with baseline on CD8^+^ T cells and normalized by day 8 (*P* < 0.001; Figure 5).

### DC and NK Cell Activation

**Figure 6:**
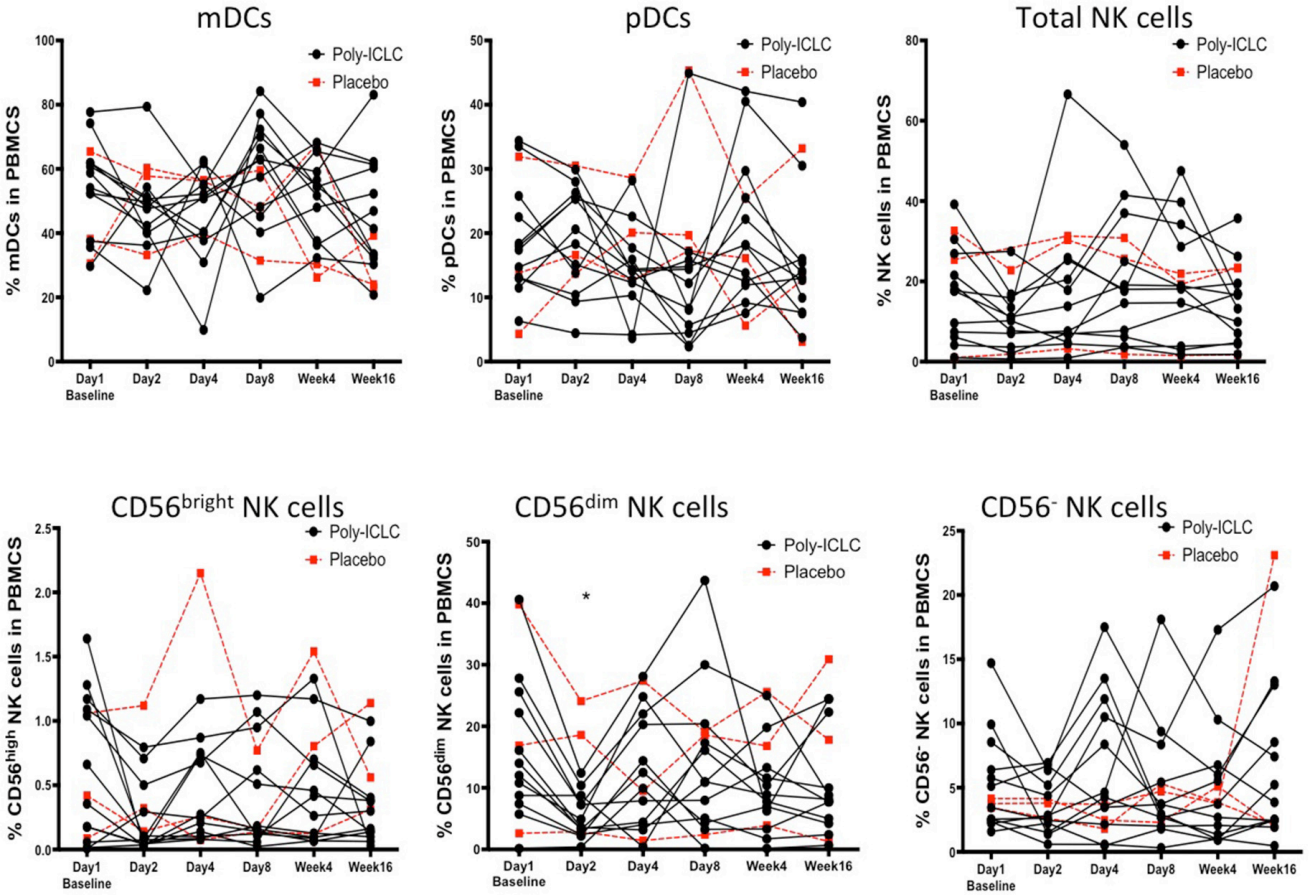
No changes in DC and NK cell numbers and activation following Poly-ICLC treatment. DC and NK cell subsets were enumerated as described following Poly-ICLC vs. placebo. Percentage of mDCs, pDCs, total NK cells, CD56 bright, CD56 dim, and CD56 negative cells in PBMCs for each subject are shown over the course of the study as measured by flowcytometry. Values for subjects in Arm A (Poly-ICLC) and Arm B (Placebo) are depicted by solid and dashed lines, respectively. Though multiple NK cell subsets declined following Poly-ICLC, the only CD56 dim NK cells were found to be statistically significant. (^*^p < 0.05, FDR <1).

Many different subsets of DCs have been identified such as CD141^+^ cDC1s, CD1c^+^cDC2s, Langerhans cells, pDCs, and inflammatory DCs. We focused on analyzing the effect of Poly-ICLC on pDCs and mDCs (most similar to cDC2) as these two are the most abundant DC subsets in blood. DCs were identified by a lack of lineage marker expression (CD3, CD14, CD16, CD20, and CD56) and positive for HLA-DR (a heterodimeric MHC-II cell surface receptor). pDCs and mDCs were identified by mutually exclusive expression of CD123 and CD11c, respectively (16, 50). NK cells were identified as being negative for CD3 and by co-expression of CD16 and CD56. CD16^+^CD56^dim^ NK cells are considered mature NK cells that are highly cytolytic and inflammatory while CD16^+/−^CD56^bright^ NK cells are highly proliferative and known for producing high levels of IFNγ upon stimulation. CD16^+^CD56^−^ NK cells have been previously described in healthy cord blood samples and chronic HIV donors (51, 52), however it is unclear if these are immature, dysfunctional NK cells or an artifact due to contamination from the myeloid fraction. There was no significant difference in percentage of pDCs and mDCs in circulation post Poly-ICLC treatment. The CD56^dim^ NK cell subsets appeared to transiently decrease on day 2 compared with baseline but subsequently returned to baseline by day 8 (*P* < 0.05; Figure 6).

To determine the phenotype of circulating DCs expression of co-stimulatory cell surface proteins CD40, CD83, CD86, all of which are required for optimally engaging and activating T cells, was examined (8, 16). A unique array of activating and inhibitory receptors control NK cell effector functions by either recognizing the presence or absence of their ligands on target cells. For our study we analyzed the expression of two activating receptors, NKp44 and NKG2D and two inhibitory receptors, TIM3, and KIR3DS1/DL1 on circulating NK cells (53, 54). The activation status of both, DCs and NK cells, remained overall unchanged from baseline. There were also no changes in intracellular cytokine secretion of TNFα, IFNα, or IL-12 observed in DCs from baseline (not shown).

### HIV-1 Latency and Reservoirs

**Figure 7:**
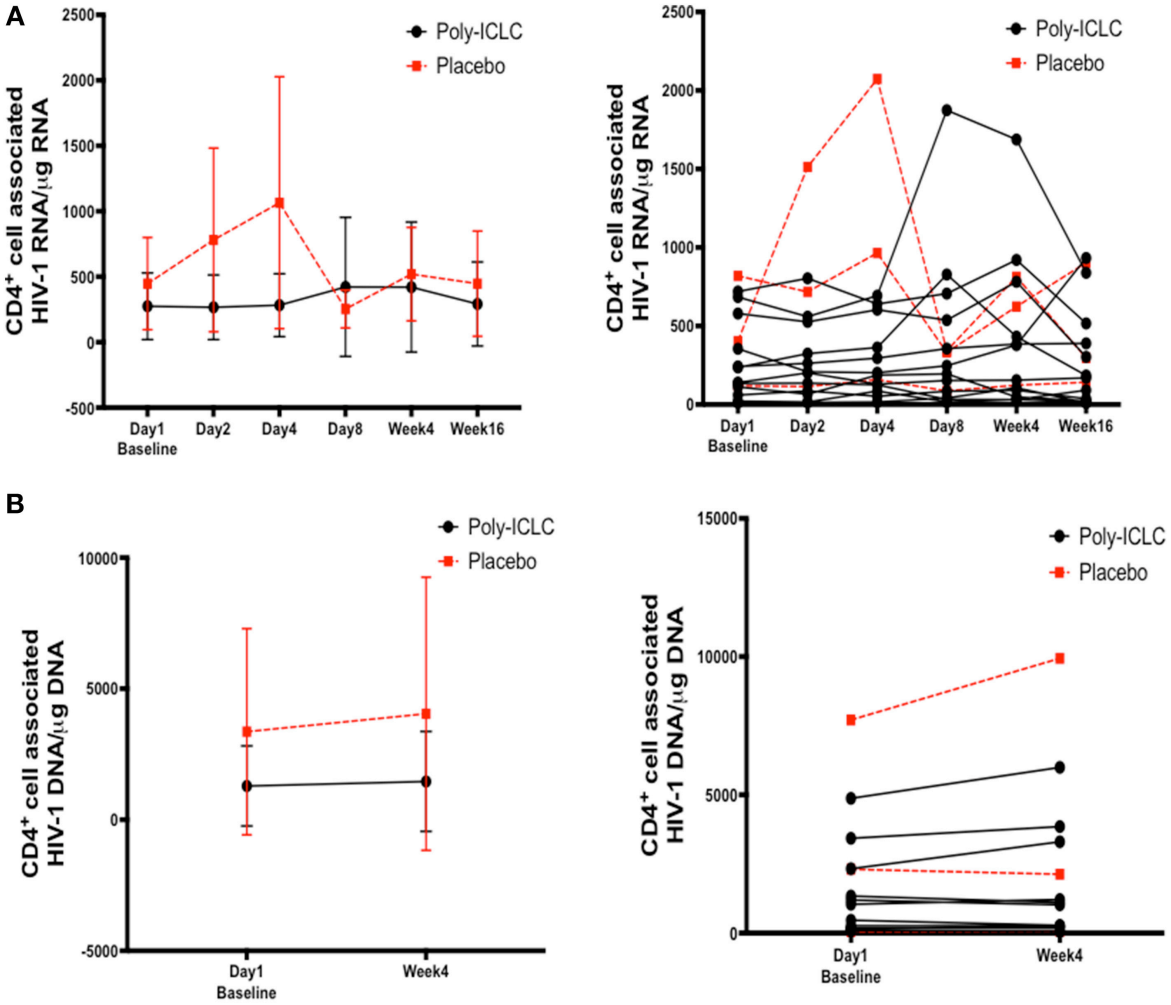
Longitudinal assessment of cell associated HIV-1 RNA and DNA values show no significant changes overall following discrete dosing of Poly-ICLC. **(A)** Following RNA extraction from purified CD4^+^ T cells at baseline and multiple time points following Poly-ICLC, rtPCR for HIV-1 RNA was performed in quadruplicate. Cell-associated HIV-1 RNA copy number is expressed as copy number per 1 μg of RNA in purified CD4^+^ T cells. The left graph depicts pooled values from all participants in Arm A (Poly-ICLC) and Arm B (placebo), with the mean values and standard deviation. The right graph depicts this data as individual values over time for each participant in Arm A and Arm B. **(B)** Following DNA extraction from purified CD4^+^ T cells at baseline and at week 4, rtPCR for HIV-1 DNA was performed in quadruplicate. Cell-associated HIV-1 DNA copy number is expressed as copy number per 1 μg of DNA in purified CD4^+^ T cells. Left graph depicts pooled values from all participants in Arm A and Arm B. The right graph shows individual values for each participant in Arm A and Arm B. Values for subjects in Arm A (Poly-ICLC) and Arm B (Placebo) are depicted by solid and dashed lines, respectively.

CD4^+^ T cell-associated HIV-1 RNA was measured to determine changes in HIV latency from baseline. Though certain individuals experienced increases in cell-associated HIV RNA following administration of Poly-ICLC at various time points, these were low in magnitude and overall was not a statistically significant finding (Figure 7A). Cell-associated HIV-1 proviral DNA was measured as an estimate of reservoir size at baseline and week 4, and there was no observed difference in this measure (Figure 7B).

## Discussion

The innate immune environment during vaccination plays a crucial role in the induction of strong and durable immunity. Here, we report multiple outcomes of the first study of Poly-ICLC, a TLR3 agonist, during treated HIV infection. In a previous study of eight healthy volunteers who received a single dose of Poly-ICLC (1.6 mg SQ), majority of participants had reported moderate or severe ISRs, along with a high frequency of systemic AEs (31). However, in our study, Poly-ICLC was very well-tolerated in the HIV-infected population studied; with AEs primarily consisting of mild ISRs in nearly all subjects and less frequent fevers and other systemic symptoms. It is unclear as to why the AEs were relatively mild in our study. It has been suggested that there may be some Poly-ICLC lot variability in terms of reactogenicity on the other hand it is also possible that dampening of innate immune responses during HIV infection may be responsible for this outcome (6, 39, 40). Though favorable adjuvant tolerability during HIV infection is advantageous, it remains unclear what effect, if any, this may have on its potency when administered with a vaccine.

Nevertheless, we observed strong upregulation of numerous ISGs and increases in circulating levels of IP-10. These responses were transient, generally peaking by 24–48 h after injection and returning to baseline by day 8. In previous studies also, the transcriptional responses following Poly-ICLC, in healthy volunteers (31) and cancer patients (28), reported transient upregulation of ISGs with accompanying induction in other innate immune pathways but to a lesser extent. Similarly, in addition to ISGs, we detected transcriptional upregulation of other innate immune pathways but did not find associated significant increases in circulating pro-inflammatory cytokines beyond IP-10. It remains unclear if a lack of secreted inflammatory signature in our study was a result of a diminished effect of Poly-ICLC during HIV infection or due to low dosing over a short period of time.

Poly-ICLC administration also did not induce activation of circulating DCs and NK cells, as gleaned from flowcytometry analysis. However, local effects on tissue DCs and other innate immune cells were likely induced as evidenced by ISRs in the vast majority of individuals. Furthermore, we observed a transient decrease in circulating NK cells subsets (CD56^dim^) following Poly-ICLC treatment, which may possibly reflect trafficking of this subset of NK cells to the tissues. In preclinical studies, Poly-ICLC has been shown to secondarily activate NK cells to enhance their cytotoxicity which may in turn augment the killing and elimination of latently infected cells alone (25, 55) or in combination with other TLR ligands (56).

Prior trials have primarily evaluated the formation of antigen-specific adaptive immune responses in patients administered with Poly-ICLC in combination with either tumor associated peptides or specific targeting antibodies (29, 30, 32, 57, 58), thus making comparison to the current study of Poly-ICLC-alone difficult. Specially given our focus on induction of innate immune responses. In future trials, vaccination with Poly-ICLC combined with HIV related peptides will serve as the true test of its potential role as an adjuvant during HIV infection.

An ongoing safety concern associated with the use of novel adjuvants in HIV-infection is the potential for induction of generalized immune activation that could increase cellular targets of infection and contribute to immunopathogenesis. Therefore, in addition to monitoring viral parameters, we gauged levels of generalized immune activation and exhaustion on CD4^+^ and CD8^+^ T cells via flowcytometry and measured circulating markers of inflammation including D-dimer, CRP, sCD14. We did not find any upregulation in markers of immune activation on CD4^+^ T cells that would lead to increased risk for infection. Similarly, markers of immune activation and exhaustion were not found to be significantly upregulated in plasma or on CD8^+^ T cells except for a transient increase in CD38, a maker of T cell activation (59), expression on CD8^+^ T cells on day 4 that returned to baseline by day 8. The elevated levels of CD38 on CD8^+^ T cells in this study were likely a result of temporary increases in IFNs induced by Poly-ICLC (60). Given the fleeting nature of this upregulation, along with the stability of other surface markers including HLA-DR, PD-1, and CTLA-4, it is unlikely that deleterious effects on adaptive immune function are imparted overall.

TLR ligands may not only serve as vaccine adjuvants during HIV infection but may also impact viral latency (17–21). Indeed, TLR ligand driven activation of innate immune cells induces inflammatory cytokines, several of which are known to promote viral reactivation (61, 62). Here, we observed no overall effect within the Poly-ICLC-treated group on viral latency via measurement of CD4^+^ T cell-associated HIV-1 RNA, despite increases detected in certain individuals following Poly-ICLC treatment that remains of unclear significance. Furthermore, there was no significant change in CD4^+^ T cell-associated HIV-1 proviral DNA, suggesting that HIV reservoirs were largely unchanged following Poly-ICLC administration. Larger studies, possibly with more frequent dosing of poly-ICLC will be needed to assess whether this adjuvant will ultimately impact the HIV reservoir. These findings are in contrast to preclinical studies with TLR ligands during HIV/Simian immunodeficiency virus (SIV) infection *in vivo* and *in vitro* (18, 21, 63). TLR9 agonist (MGN1703) has been shown to enhance HIV-1 transcription *in vitro* in PBMCs from aviremic donors (18). Moreover, recent preclinical studies of TLR7 agonists (GS-9620 and GS-980, Gilead) in SIV-infected non-human primates receiving cART, revealed transient increases in plasma SIV RNA levels and decreased viral reservoirs in PBMCs and lymph nodes (21). Additionally, viral load in two of the treated animals remained undetectable for more than 90 days after ART interruption (21).

There remains scant clinical data regarding the use of TLR ligands alone during HIV infection with the exception of a clinical trial that administered TLR9 agonist MGN-1703, twice weekly for 4 weeks, in cART suppressed patients (20). The study demonstrated a significant increase in plasma viremia in 6 out 15 patients along with increase in pDC, NK cell, and CD8^+^ T cell activation and induction of plasma IFNα, TNFα, IFNγ during the course of the study. The differential findings in these studies with other TLR ligands vs. the current trial may be related to distinct effects of the TLR ligands themselves and to the dissimilar dosing strategies utilized. Though each of these TLR ligands (3, 7, 9) are involved in anti-viral immunity and known to stimulate type I IFN responses, TLRs 7 and 9 are expressed on pDC, as opposed to TLR3 which is expressed on mDC, perhaps accounting for divergent effects of these ligands on innate immunity in a vaccine context (64). Moreover, latency reversal effects of TLR7 ligands in SIV infection setting was not observed following the first two doses, but became evident after 3 more doses of the 10–19 received in total, indicating a net cumulative effect (21). It is possible that additional and/or higher doses of Poly-ICLC would have resulted in similar effects on viral latency. Given the early phase of this clinical trial with a primary emphasis on safety, such dosing regimens were not suitable. Exploration of increased or expanded dosing strategies and routes of administration in future studies will help clarify Poly-ICLC's effect on HIV latency.

There are several limitations to this study that must be considered, including relatively small sample size. This prevented the evaluation of multiple sub-group analyses, including the influence of individual ART regimens on immunologic and virologic parameters, which have been shown to alter TLR responsiveness *in vitro* (65). Other limitations include the overall homogeneity of the study population in terms of gender, though racially and ethnically diverse. Furthermore, due to strict study entry criteria designed to maximize safety, the exclusion of older individuals (>55 years) and common comorbidities such as cardiovascular disease and diabetes further limit the generalizability of the safety findings in the HIV-infected population as a whole. Additional studies that increase the diversity of the HIV-infected individuals included will help to bolster these safety findings.

In summary, the TLR3 ligand, Poly-ICLC, was safe and tolerable in the HIV-infected population studied and transiently stimulated innate immune responses without resulting in undue immune activation. Given the promising safety profile of PolyICLC in our trial, future studies may now be designed; enlisting a larger cohort, with a wider inclusion criteria, longer duration, and more frequent dosing to further optimize the potential use for PolyICLC as a latency reversal agent. Moreover, this study paves the way for HIV therapeutic vaccines trials that combine Poly-ICLC with HIV antigen vaccines to enhance adaptive immunity to ultimately reduce viral reservoirs.

## Ethics Statement

This study was carried out in accordance with the good clinical practice (GCP), with written informed consent from all participants in accordance with the Declaration of Helsinki. The protocol was approved by Ethics Committees and IRBs at Icahn School of Medicine at Mount Sinai and Rockefeller University Hospital.

## Author Contributions

MS: critical data analysis, manuscript writing, reviewing, and editing. RS and EM: protocol immunologists. RS and HD: specimen handling and processing at Vaccine and Cell Therapy Laboratory at Icahn School of Medicine at Mount Sinai. HM: protocol virologist and manuscript review. ML: data and regulatory management. JC: biostatistician. AS: study design and reagents. MM: co-principal investigator, manuscript revision, and study design. NB and EM: co-principal investigators, senior authors, study design, data interpretation and manuscript writing and reviewing.

### Conflict of Interest Statement

NB has received research funds from Merck and is on the senior advisory board of Check Point Diagnostics, Curevac, Prime vax, Neon, and Tempest Therapeutics. EM is a current employee of Regeneron Pharmaceuticals, and serves on the Board of Trustees of Abzyme Research Foundation. AS is the CEO and Scientific Director of Oncovir, Inc. RS is a current employee of Genentech. The remaining authors declare that the research was conducted in the absence of any commercial or financial relationships that could be construed as a potential conflict of interest.

## Supplementary Material

Supplemental Figure 1 No induction of soluble mediators of inflammation upon Poly-ICLC administration. Plasma levels of IFN alpha, IFN beta, TNF alpha, sCD14, D-dimer, and C-reactive protein (CRP) for each subject are shown as a mean with standard deviation over the course of the study as measured by ELISA. Values for subjects in Arm A (Poly-ICLC) and Arm B (Placebo) are depicted by solid (black) and dashed (red) lines, respectively. No longitudinal changes were found to be statistically significant following Poly-ICLC administration.

Supplemental Table 1 Transient upregulation of pro-inflammatory genes in subjects whom received Poly-ICLC. Transcriptional responses were evaluated longitudinally in subjects' PBMCs using NanoString Technologies (nCounter® gene expression panel, human inflammation kit). Fold change (FC) from baseline of all significantly up regulated genes (*p* < 0.05, FDR < 1, FC ≥ +/−1.5) from subjects in Arm A (Poly-ICLC) are shown through day 8. No significant changes were observed in subjects' PBMCs in Arm B (Placebo) (not shown).
