# Supplementary figures and images for: Poly-ICLC, a TLR3 Agonist, Induces Transient Innate Immune Responses in Patients With Treated HIV-Infection: A Randomized Double-Blinded Placebo Controlled Trial
Source: Front Immunol. 2019 Apr 9;10:725. doi: 10.3389/fimmu.2019.00725 (PMC6467168; doi:10.3389/fimmu.2019.00725)

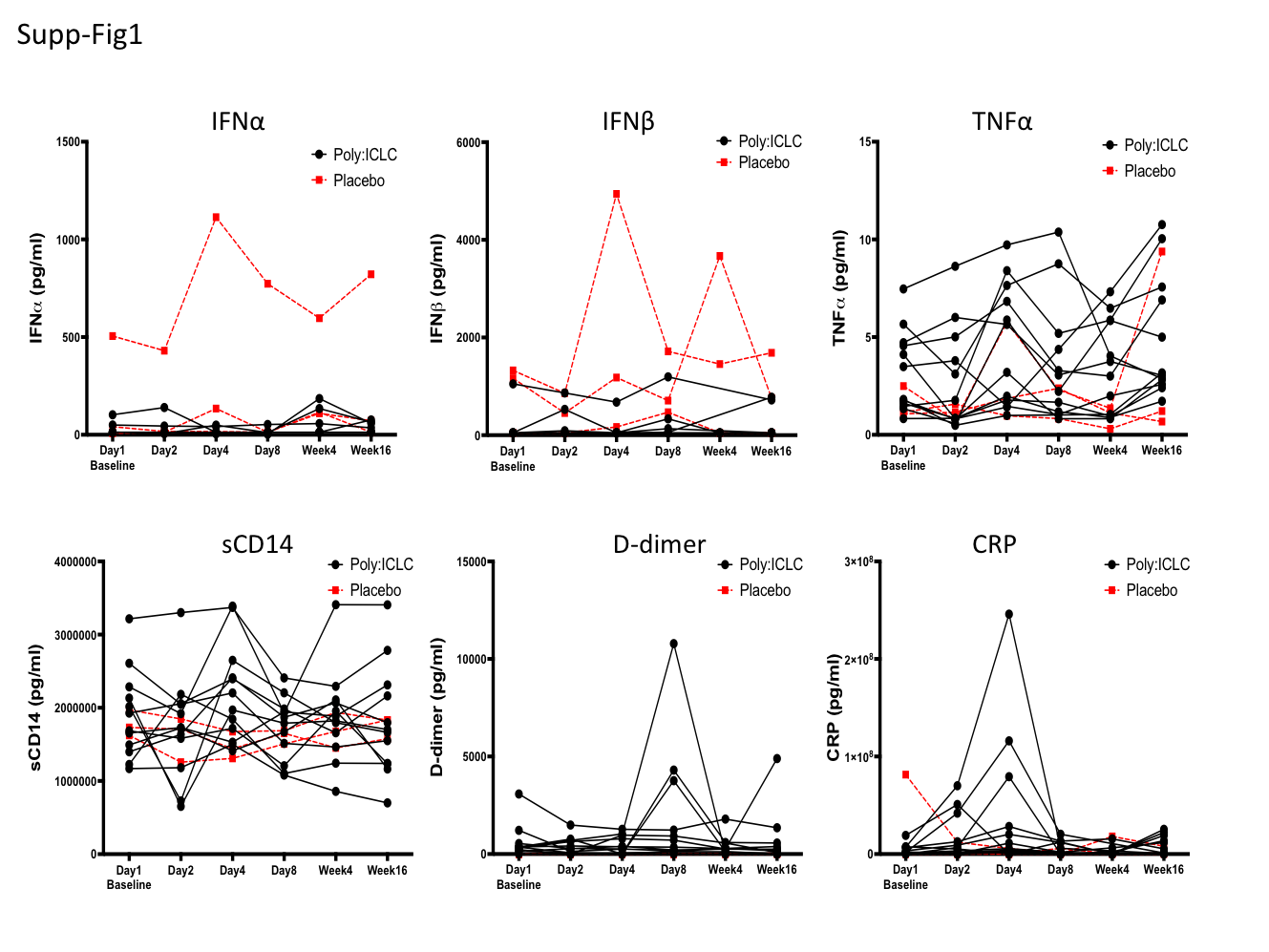

Supplement: Supplemental Figure 1 — No induction of soluble mediators of inflammation upon Poly-ICLC administration. Plasma levels of IFN alpha, IFN beta, TNF alpha, sCD14, D-dimer, and C-reactive protein (CRP) for each subject are shown as a mean with standard deviation over the course of the study as measured by ELISA. Values for subjects in Arm A (Poly-ICLC) and Arm B (Placebo) are depicted by solid (black) and dashed (red) lines, respectively. No longitudinal changes were found to be statistically significant following Poly-ICLC administration. [file Image_1.TIFF]
